# Drug Screening and Drug Repositioning as Promising Therapeutic Approaches for Spinal Muscular Atrophy Treatment

**DOI:** 10.3389/fphar.2020.592234

**Published:** 2020-11-12

**Authors:** Giovanna Menduti, Daniela Maria Rasà, Serena Stanga, Marina Boido

**Affiliations:** Department of Neuroscience Rita Levi Montalcini, Neuroscience Institute Cavalieri Ottolenghi, University of Turin, Turin, Italy

**Keywords:** survival motor neuron, motor neuron disease, therapy, cell death and degradation, mitochondria, cytoskeleton dynamics, neurotransmitter modulation, neuromuscular junction stabilization

## Abstract

Spinal muscular atrophy (SMA) is the most common genetic disease affecting infants and young adults. Due to mutation/deletion of the survival motor neuron (*SMN*) gene, SMA is characterized by the SMN protein lack, resulting in motor neuron impairment, skeletal muscle atrophy and premature death. Even if the genetic causes of SMA are well known, many aspects of its pathogenesis remain unclear and only three drugs have been recently approved by the Food and Drug Administration (Nusinersen—Spinraza; Onasemnogene abeparvovec or AVXS-101—Zolgensma; Risdiplam—Evrysdi): although assuring remarkable results, the therapies show some important limits including high costs, still unknown long-term effects, side effects and disregarding of *SMN*-independent targets. Therefore, the research of new therapeutic strategies is still a hot topic in the SMA field and many efforts are spent in drug discovery. In this review, we describe two promising strategies to select effective molecules: drug screening (DS) and drug repositioning (DR). By using compounds libraries of chemical/natural compounds and/or Food and Drug Administration-approved substances, DS aims at identifying new potentially effective compounds, whereas DR at testing drugs originally designed for the treatment of other pathologies. The drastic reduction in risks, costs and time expenditure assured by these strategies make them particularly interesting, especially for those diseases for which the canonical drug discovery process would be long and expensive. Interestingly, among the identified molecules by DS/DR in the context of SMA, besides the modulators of *SMN2* transcription, we highlighted a convergence of some targeted molecular cascades contributing to SMA pathology, including cell death related-pathways, mitochondria and cytoskeleton dynamics, neurotransmitter and hormone modulation.

## Introduction to Spinal Muscular Atrophy and Available Therapies

### Spinal Muscular Atrophy Pathogenesis

Spinal muscular atrophy (SMA) is a severe neuromuscular disorder affecting children and young adults with an incidence of one in 3,900–16,000 live births. In Europe, 4,653 patients were genetically diagnosed between 2011 and 2015, with 992 diagnosed in 2015 alone (Verhaart et al., 2017). SMA is characterized by brainstem and spinal motor neuron (MN) degeneration, due to mutation/deletion of survival motor neuron 1 (*SMN1*) gene. In physiological conditions, the encoded SMN protein has many important roles such as in the assembly of the spliceosome, biogenesis of ribonucleoproteins, mRNA trafficking and local translation, cytoskeletal dynamics, cellular bioenergetics, endocytosis and autophagy (for an extensive review on SMN functions, see Chaytow et al., 2018). In SMA, its lack determines motor impairment, muscle atrophy and premature death. However, the ubiquitous deficiency of SMN protein leads to consider SMA a multisystemic disorder, since its depletion can dramatically affect many other organs/systems (including heart, pancreas and immune system) (Bottai and Adami, 2013).

More in details, in humans there are two *SMN* genes i) the telomeric form *SMN1*, which translates for a ubiquitous protein (full-length SMN or FL-SMN), and ii) its centromeric homologous *SMN2* which mostly generates a truncated and rapidly degraded protein delta7-SMN (SMNΔ7) and only about 10% of FL-SMN (Lorson et al., 1999). Therefore, in SMA, the production of functional SMN protein depends only on *SMN2* gene and the degree of the disease severity is based on *SMN2* copy number. Indeed, there are four types of SMA (reviewed by D’Amico et al., 2011; Boido and Vercelli, 2016; Vaidya and Boes, 2018). SMA 1, also known as Werdnig-Hoffmann disease, is diagnosed within 6 months of age: it is the most severe and the most common type (60% of all SMA cases) and it is generally fatal early on in life. SMA one babies show severe muscle weakness and trouble breathing due to spared diaphragm and feeble intercostal muscles; they also have difficulties in coughing, swallowing and feeding. SMA two is usually diagnosed between 6 months and 2 years of age, and the life expectancy is reduced. SMA type 2 babies show significant delay in reaching motor milestones or fail to meet milestones entirely: they can sit up without help, though they may need assistance getting into a seated position, but they are unable to walk and require a wheelchair. SMA 3, also called Kugelberg-Welander disease or juvenile SMA, is usually diagnosed after 18 months of age. Individuals affected by SMA three can be divided in two subgroups depending on the disease onset: i) patients with onset before 3 years of age are initially able to walk, but have increasingly limited mobility as they grow due to scoliosis and many need to use a wheelchair; and ii) patients with onset after 3 years of age might continue to walk and show slight muscular weakness. Finally, SMA type 4 is very rare and usually appears in adulthood (after 18 years of age), leading to mild motor impairment and no respiratory and nutritional problems.

### Spinal Muscular Atrophy Approved Drugs

Despite the disease severity and its well-known genetic causes, until 2017 no treatment was available for SMA. Indeed, the efforts of the scientific, pharmaceutical, academic and clinical communities led to the discovery of effective drugs able to restore *SMN1* or to increase the expression of *SMN2* gene, in order to compensate the lack of FL-SMN protein.

Nusinersen (Spinraza) from Biogen is the first drug approved by the Food and Drug Administration (FDA) (in December 2016) and by the European Medicines Agency (EMA) (in June 2017) for both infants and adults with SMA. It is a modified 2′-O-methoxyethyl antisense oligonucleotide (ASO) designed to increase the expression of the SMN protein (Chiriboga et al., 2016). Nusinersen increments the capability of *SMN2* to produce FL-SMN by binding to the intron-splicing silencer region N1 in the *SMN2* pre-messenger RNA (pre-mRNA) promoting exon seven inclusion (Singh et al., 2006). Since the drug cannot pass through the blood brain barrier (BBB), it must be intrathecally administered. On May 2019, another drug, AVXS-101 (Zolgensma) from AveXis, a Novartis company, has been approved by the FDA, after the publication of the positive results of the phase one study called START (Identifier: NCT01547871) on its safety and efficacy after a one-time infusion in SMA one patients with symptoms before 6 months of age. In March 2020, it also received a conditional marketing authorization and it has been approved in May 2020 from EMA (Zolgensma, 2020a; Novartis, 2020).

AVXS-101 is the non-replicating recombinant AAV9 containing the complimentary DNA of the human *SMN* gene under the control of the cytomegalovirus enhancer/chicken-β-actin-hybrid promoter. The phase 3, open-label, single-arm and single-dose study delivering AVXS-101 by intravenous infusion called STR1VE (Identifier: NCT03306277) has been concluded in November 2019. On March 2020, the company showed the results of the concluded study STR1VE-US: “nine of 22 patients in the completed pivotal study demonstrated the ability to thrive, a stringent composite endpoint remarkable compared to untreated children with SMA type 1; the study showed that patients achieved rapid and sustained improvement in motor function” (for the complete press-release see Zolgensma, 2020b). Up to now, different studies are still ongoing: START Long Term Follow Up (Identifier: NCT03421977) aims to estimate the long-term safety on patients who completed the study START; whereas SPR1NT (Identifier: NCT03505099), a phase 3 open-label, single-arm, multi-center trial has been designed to evaluate the safety and efficacy of a one-time intravenous infusion in pre-symptomatic patients with SMA 1. The overall expected advantage of the AAV9 is that by a single administration patients could have a systemic and long-term lasting expression of *SMN1* (Al-Zaidy et al., 2018). A recent review, which compared all the published data and clinical trials on AVXS-101, confirmed that it represents an effective therapy for younger pediatric patients with SMA 1 (Stevens et al., 2020).

Risdiplam (Evrysdi), from Genentech, a member of the Roche Group, has been approved by the FDA on August 7th, 2020 for the treatment of children from 2 months of age on of adult (FDA Approves Genentech’s Evrysdi (risdiplam) for Treatment of Spinal Muscular Atrophy (SMA) in Adults and Children 2 Months and Older, 2020). Risdiplam is a mRNA splicing modifier which increases SMN protein expression. It is a liquid medicine, which can be administered orally without the need for hospitalization. Risdiplam is currently under study in four open-label trials: i) FIREFISH (Identifier: NCT02913482) and ii) SUNFISH (Identifier: NCT02908685) to investigate safety, tolerability, pharmacodynamics and kinetics and Efficacy in SMA 1 or SMA2 and 3, respectively; iii) JEWELFISH (Identifier: NCT03032172) and iv) RAINBOWFISH (Identifier: NCT03779334) to reveal the long-term efficacy of Risdiplam in patients who previously received another SMA treatment and infants from birth till 6 weeks of age, respectively.

Besides the ascertained data reporting the safety profile of these treatments and their significant benefits for some cohorts of SMA patients, it is necessary to take into account also their limitations, not only from the medical point of view but also from the socio-economical side.

### Limits of Current Therapies and the Need of New Targets

One important limit of the current available treatments for SMA is that such approaches are merely *SMN*-dependent strategies and overlook other molecular pathways contributing to SMA pathogenesis (see beyond). To overcome this problem, combinatorial therapies should be considered, in order to redefine the timing and parameters of administration of the *SMN*-enhancing therapies currently in use, and to consider the synergistic effects with other drugs. Overall, combinatorial treatment strategies are required to face the *SMN*-independent features of SMA pathology. Moreover, the efficacy of the available treatments strictly depends on the age/type of patients: indeed, the effects induced by *SMN*-enhancing therapies are most consistent in the early-treated patients, whereas delayed interventions lead to less efficient or none rescue of motor neuron defects (Hoolachan et al., 2019; Hensel et al., 2020; Poletti and Fischbeck, 2020). In fact, SMN-restoring approaches seem particularly effective when the MNs are still alive and muscle functions not irreversibly compromised, as in the early phase of SMA disease. Depending on the age at the beginning of the therapy, Nusinersen and AVXS-101 can significantly extend the survival of babies with SMA 1, allowing motor milestone achievement; similarly, young patients with SMA two also show progresses on different motor scales after treatment. However, when Nusinersen is administered in adults patients with SMA type 2 and 3 (20–68 years old), improvements did not reach similar significant levels and could just support the stabilization of motor functions and the reduction in the symptom worsening (Jochmann et al., 2020; Mercuri and Sansone, 2020). While on one hand this piece of evidence strongly encourages the improvement of newborn screening methods, on the other hand it explains the growing pressure from late-symptomatic patients and caregivers for accessing to additional treatments (Richelme, 2019; Ramdas and Servais, 2020). Furthermore, prospective studies with larger patient numbers as well longer follow-up durations are required to better define the safety and efficacy of the treatments (Lee et al., 2019; Malone et al., 2019; Zuluaga-Sanchez et al., 2019).

To note, the current therapies present important limits such as i) the invasiveness of the administration route (in the case of Nusinersen), ii) more or less severe side effects, and iii) their cost and commercial accessibility. Indeed, Nusinersen administration requires hospitalization since it is administered intrathecally at least three times per year, for the entire life of the patient. Moreover, SMA patients generally develop severe scoliosis and spinal deformities, which in turn complicate or hinder this way of administration. These limitations can be circumvented by the development of systemically (as AVXS-101) or orally administered drugs able to cross the BBB (as Risdiplam) (Poletti and Fischbeck, 2020). To date, other orally delivered compounds are in final phases of clinical development and trials (Ramdas and Servais, 2020): these include a mRNA splicing corrector, branaplam (LMI070, Novartis), and a fast-skeletal muscle troponin activator, reldesemtiv (CK-2127107, Cytokinetics) (Rao et al., 2018). These alternative administration routes can also assure peripheral SMN-restoration, complementing the SMN central effects.

Additionally, the available treatments can also cause important side effects such as headache, back pain (Nusinersen), acute liver damage, bleeding, and heart damage (AVXS-101) (Gidaro and Servais, 2019). Moreover, about 5% of AVXS-101-treated patients can develop anti-AAV9 antibodies (viral titer greater than 1:50) this can increase the risk for immune response to gene therapy and reduce its therapeutic benefit (Al-Zaidy and Mendell, 2019). Finally, Nusinersen and AVXS-101 are listed among the most expensive drugs in the world: the relative cost-effectiveness ratio data reports, constantly updated, must be considered (Rao et al., 2018; Hoot, 2019; Malone et al., 2019).

Considering the limitations of the approved drugs for SMA, it is evident that the search for other potential therapies is necessary.

Below, we firstly describe two common methods to find new drugs: drug screening (DS) and drug repositioning (DR). Secondly, we make a comprehensive review of DS and DR studies conducted specifically in the SMA field, describing procedures, models, drug-targeted signaling pathways and results.

## Drug Screening and Drug Repositioning for SMA

**FIGURE 1:**
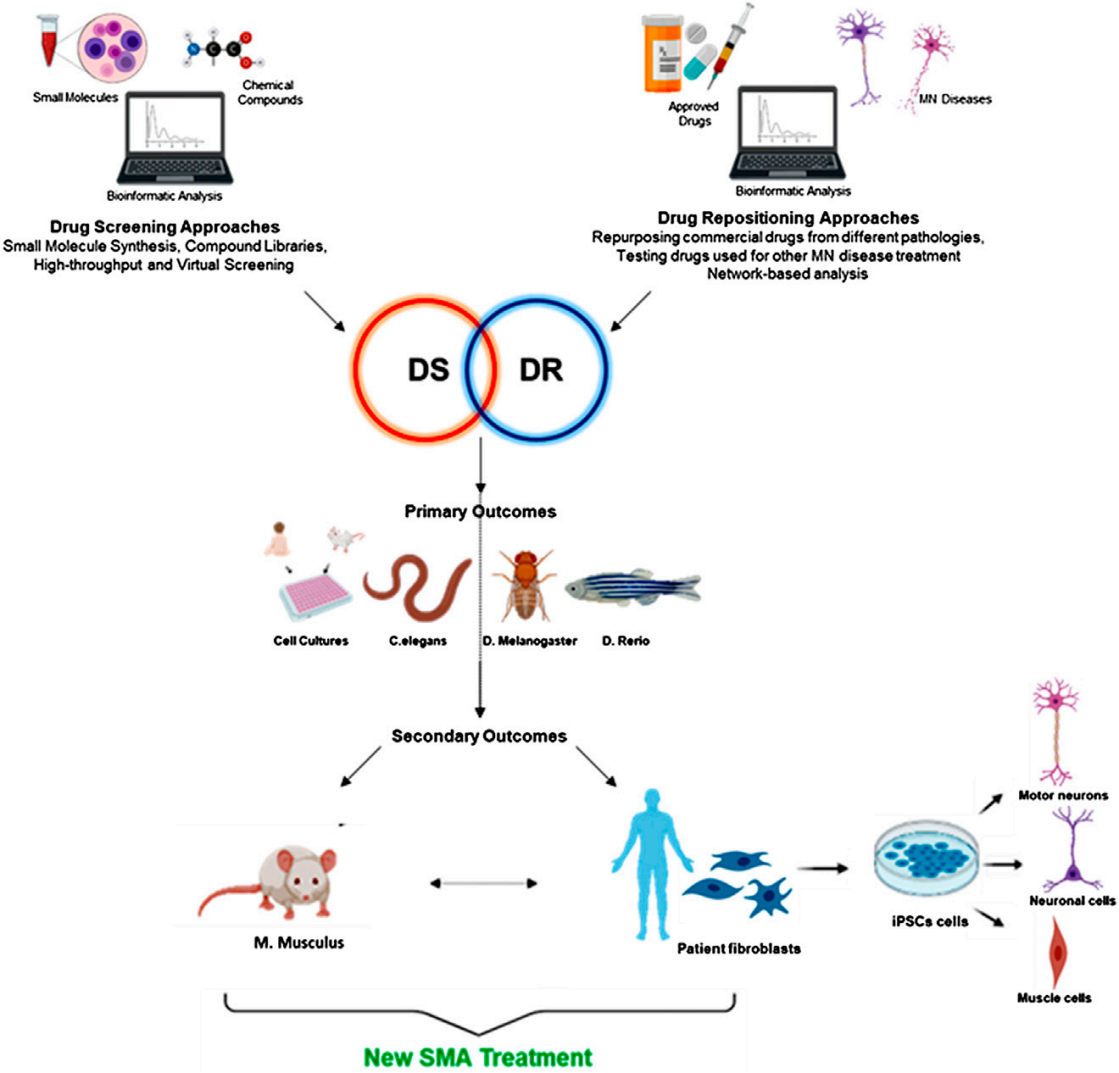
DS and DR methods as powerful approaches in SMA therapeutic research. The two approaches, eventually in combination, can pave the way for rapid identification of drugs for novel SMA treatments. The compounds (present in large screening libraries) can be tested for primary outcomes (continuous arrow, in the middle) firstly on “simplified” SMA models (cell cultures and/or invertebrates models), and then on SMA mice and/or patient-derived iPSCs (differentiated in neurons, MNs and muscle cells, eventually cocultured) to achieve secondary outcomes. In some cases (dotted arrow, in the middle) the hit compounds can be directly tested on murine models and iPSCs. Created with BioRender software. DR, drug repositioning; DS, drug screening; MN, motor neuron; SMA, spinal muscular atrophy.

DS is a process by which a huge amount of compounds can be relatively quickly tested and selected as effective, by means of appropriate experimental models. On the contrary, DR (also currently referred as drug repurposing, reprofiling, retasking, or therapeutic switching) consists in a strategy to attribute new uses to drugs (generally already FDA approved) that are outside the scope of the original medical indications reducing risks and costs associated with time consuming new drug development programs. Briefly, the two approaches, eventually in combination, exploit the availability of large compound libraries, which include thousands of chemical and natural compounds and/or FDA-approved substances: Prestwick Chemical Library, MicroSource Discovery Systems, ComGenex, National Institute for Neurological Disorders and Stroke, TimTec, IBS, and ChemBridge are just few libraries that have been used to find new SMA therapies during the last years (Kelley et al., 2004; Sleigh et al., 2011; Konieczny and Artero, 2020). Afterward, once screened in search of specific cellular/molecular readouts, the substances can be firstly tested on “simplified” SMA models (cell cultures and/or invertebrates models), and then (or directly, in some cases) on SMA mice and/or patient-derived iPSCs (Figure 1).

Many kinds of DS approaches exist since a long time, including High-throughput, Focused, Fragment, Structural aided drug design, Virtual, Physiological, and Nuclear Magnetic Resonance screens (for an extensive review, see Hughes et al., 2011). These methods can be also extended to DR research, when FDA-approved drugs (already employed for the treatment of other pathologies) are investigated. Notably, High-throughput, Virtual and Physiological screenings are those ones mainly used in the SMA field.

High-throughput and Virtual screening are two rapid methods that allow wide-scale assays (by omics studies), in particular during the first screening phase to identify hit-compounds. As reviewed in Fox et al., (2006), to date, the High-throughput approaches, based on phenotypic screening of entire compound libraries, are commonly used in drug discovery processes, including different phases: target validation, assay development, secondary screening, ADME/Tox, and lead compound optimization (Fox et al., 2006). As an example, in SMA drug discovery, the 3,6-disubstituted pyridazine was identified through High-throughput screening in the NSC-34 cell line containing a *SMN2* minigene reporter, and was then chemically modified leading to the synthesis of Branaplam (nowadays in clinical trial phase 2 for SMA treatment) (see section *Direct and Indirect Modulation of Survival Motor Neuron 2 Transcription*) (Cheung et al., 2018). On the other hand, virtual screening methods (such as ligand-based/structure-based virtual screening; as reviewed by McInnes, 2007), which exploit docking of new compounds that could bind known targets, are widely and successfully used to identify novel drugs for the treatment of neurodegenerative diseases (Makhouri and Ghasemi, 2017). Although these virtual screening paradigms have been poorly carried out in the SMA field, some successful applications of these methods have been reported: in particular, molecular docking studies highlighted both the binding mode of the C5-substituted quinazolines against a RNA metabolism regulator (the scavenger decapping enzyme, DcpS), and the binding mode of E-resveratrol, suberoylanilide hydroxamic acid and valproic acid against HDAC8 (see section *Direct and Indirect Modulation of Survival Motor Neuron 2 Transcription*) (Dayangaç-Erden et al., 2008; Singh et al., 2008; Makhouri and Ghasemi, 2017). Some of these compounds identified by DS (e.g., RG3039 and valproic acid) underwent clinical trial studies for SMA treatments (Kissel et al., 2011; Gogliotti et al., 2013). Moreover, molecular docking studies represent a rapid and cheap strategy to test FDA-approved drugs, paving the way to DR (Hughes et al., 2011; Pushpakom et al., 2018). Finally, as reviewed by Hughes et al., (2011), in the last stage of DS process, the Physiological screening is generally exploited to assay only hit-compounds (Hughes et al., 2011): indeed, this screening, based on tissue specific analyses and readouts (as muscular tissues in case of SMA), can require complex experimental models, such as transgenic mice (Le et al., 2005).

In the last years, DS/DR methods allowed both an in depth understanding of SMA pathological mechanisms and the identification of therapeutic approaches, including enhancing *SMN* function and regulating the *SMN* exon seven splicing. Furthermore, new targets mainly related to the rescue of the downstream effects of SMN protein depletion have been also highlighted. With this purpose, DR strategies have been applied in SMA field, allowing either the usage of established approaches on new targets, or the development of novel approaches on established targets (Ashburn and Thor, 2004). Indeed, the advantages of this approach can be summarized in three main features: i) it provides a lower risk of drug failure; ii) it reduces time frame for drug development, by skipping several clinical trial steps; and iii) it needs lower investment in clinical trials. This is due to pre-existing proofs of safety and efficacy for the repurposed drugs. In fact, the success rates for a repurposed drug to the market are significantly higher compared to specific *de novo* clinical trials (more than 15% in phases II–III clinical trial) (Hoolachan et al., 2019). For this reason, DR became a helpful approach in orphan diseases (designation granted by FDA) such as SMA (Hoolachan et al., 2019). In addition, the development costs related to DR are estimated to be thousands folds lower than those of a new chemical compound (Lotfi Shahreza et al., 2018; Pushpakom et al., 2018).

To date, albeit successfully, the process of DR has been largely opportunistic and serendipitous, often lacking a systematic approach aimed at the identification of a drug off-target or new target effects, suggesting its repositioning (Lotfi Shahreza et al., 2018; Lipinski and Reaume, 2020). During the early stages of the DR process (hypothesis generation phase), a potential repurposable drug can be identified with a high level of confidence thanks to a systematic approach and by exploiting different types of data coming from modern DS methods (i.e., computational and experimental approaches).

The computational approaches, as reviewed in detail by Lotfi Shahreza et al. (2018), involve the main *in silico* methods, based on docking simulation and machine learning (drug-based/disease-based). These methods allow to evaluate new indications on chemical ligands and protein targets or to discover repositioning opportunities (Lotfi Shahreza et al., 2018). Furthermore, as a natural evolution of these studies, promising results have been achieved in network-based computational biology, which attempts to identify pharmacological targets by reconstructing the biological network in different pathologies, simulating its interactions and highlighting correlations between drug targets (Lotfi Shahreza et al., 2018; Sam and Athri, 2019; Singh et al., 2020). In this way, the integration of “big data” generated by high-performance DNA and RNA sequencing, mass spectrometry, metabolomics and transcriptomic studies leads to the generation of different types of network-based association studies (Lotfi Shahreza et al., 2018; Pushpakom et al., 2018; Álvarez-Machancoses et al., 2020).

DR computational methods have already been exploited in the SMA field. In particular, Artero’s group using the Prestwick Chemical Library drug database (constituted by almost 1,280 compounds) (Prestwick Chemical, 2013) discovered a repurposed FDA-approved small molecule, the antibiotic Moxifloxacin, with the potential to become a new therapy for SMA (see section *Direct and Indirect Modulation of Survival Motor Neuron 2 Transcription*) (Konieczny and Artero, 2020). However, DR is a complicated process in which the computational approach *per se* is not sufficient to achieve satisfactory results. Indeed (as reviewed by Pushpakom et al., 2018; Lipinski and Reaume, 2020), the intertwining of computational and experimental methods achieves the reliability of DR approach. Data from large-scale drug screens, combined with genomic data, binding assays and High-throughput phenotypic screening, can be used to identify novel targets of known drugs (Pushpakom et al., 2018; Lipinski and Reaume, 2020). As an example, *in vivo* phenotypic screenings have been also exploited in the SMA field, allowing the discovery of small molecules able to target the RNA splicing specifically enhancing the exon seven inclusion of *SMN2* transcript and the SMN protein levels. Indeed, in 2014, Naryshkin and coll. published the first report of a phenotypic screen yielding selective *SMN2* splice modulators, leading to Risdiplam (RG7916), a recently FDA-approved drug for SMA treatment (Naryshkin et al., 2014; FDA Approves Genentech’s Evrysdi (risdiplam) for Treatment of Spinal Muscular Atrophy (SMA) in Adults and Children 2 Months and Older, 2020), while in 2015, Palacino’s group identified the splicing modulator Branaplam (LMI070) (see section *Direct and Indirect Modulation of Survival Motor Neuron 2 Transcription*) (Palacino et al., 2015). Most importantly, DR phenotypic screening allowed uncovering novel *SMN*-independent targets and drug paradigms, such as Olesoxime (see section *Mitochondria-Related Pathways*). This compound has been identified as potential therapeutic agent for both SMA and ALS (Calder et al., 2016; Swalley, 2020), since it is able to preserve mitochondrial function and protect MNs from degeneration (Calder et al., 2016; Blasco et al., 2018; Rovini et al., 2019). Indeed, a successful repositioning strategy in SMA treatment may be to identify drugs that are currently used to treat other neuromuscular diseases [such as ALS, hereditary spastic paraplegia (HSP) and Duchenne dystrophy (DD) (Patten et al., 2014)]: FDA-approved drugs modulating the pathogenetic pathways shared by these diseases have been proposed for SMA patients, as riluzole (Rilutek™ or Teglutik™) (see section *Neurotransmitters’ Modulation*), commonly administered for ALS (Calder et al., 2016; Hoolachan et al., 2019; Hensel et al., 2020). Also other drugs originally developed for different neurodegenerative diseases (such as rasalgiline and masitinib) could potentially represent promising SMA treatments (Hoolachan et al., 2019; Hensel et al., 2020).

Overall, we will focus here on the different DR and DS approaches employed to screen and identify candidate molecules for SMA treatment. Typically, *in vitro* phenotypic screenings exploit a wide range of cell-based assays in a 96-well format, including cellular disease models, highly engineered immortalized cell lines or different kind of induced Pluripotent Stem Cells (iPSCs); moreover, whole-organism phenotypic assays (using *C. elegans*, *Drosophila*, zebrafish, and mouse models) are also very important for their physiological relevance.

## Experimental Models for Drug Screening/Drug Repositioning Studies in the Spinal Muscular Atrophy Field

Over the years, several experimental models have been developed to identify and improve SMA therapies. *In vitro* models are needed for High-throughput screenings where hundreds or thousands of compounds must be tested, while animal models are more indicated for the final study phases to assess phenotypical effects of hit compounds (Figure 1).

To perform large High-throughput screenings for SMA therapy, different cell lines can be used, including NSC-34 (murine MNs), HEK-293 (human embryonic kidney cells) and C33A (human cervical squamous cell carcinoma) (Zhang et al., 2001; Sumner et al., 2003; Jarecki et al., 2005). A more recent approach consider the use of patient fibroblasts and iPSC models, allowing the possibility to tailor the medical treatments as personalized medicine (Lai et al., 2017; Wang et al., 2019).

These experimental models, to apply High-throughput screening systems, are pivotal for the rapid validation of compound libraries. For example, Jarecki and coll. used a *SMN2* promoter β-lactamase reporter gene test in NSC-34 cell line (Jarecki et al., 2005), while Zhang and collaborators developed a wide-scale screening approach based on the insertion of *SMN2*-luciferase or *SMN2*-GFP mini-gene reporters into HEK-293 and C33A (Zhang et al., 2001). These cellular models allowed to easily identify compounds that interfere with *SMN2* gene expression and, to date, *SMN2*-luciferases mini-gene reporter is the most used one (Son et al., 2019). In 2013, Letso’s group developed a High-throughput screening test, to evaluate through ELISA assays the endogenous SMN protein levels in SMA patient fibroblasts (GM03813, GM09677, and GM00232) (Letso et al., 2013). More recently, Wang and collaborators tested a small library of 980 compounds, using the HEK293 cell line in which human *SMN2*-GFP gene reporter was targeted by CRISPR/Cas9–mediated homologous recombination (Wang et al., 2019).

Cellular models can also be used to evaluate hit-compounds during the final screening phases; the most used are SMA patient fibroblast lines (GM03813, GM03814, and GM22592) (Cherry et al., 2013; Wang et al., 2019), human iPSCs-derived neurons (Lai et al., 2017) or MNs (Wang et al., 2019), and co-culture of human MNs and skeletal muscle cells (Santhanam et al., 2018). Very recently, a promising (but not yet tested for DS/DR) tool has been developed: it consists in an on-chip 3D neuromuscular junction (NMJ) model, with optogenetically controllable human iPSCs-derived MNs and skeletal muscle cells (Osaki et al., 2020).

However, in the majority of cases, the final screening phase to assess effectiveness of selected hit-compounds is carried out using animal models. The percentage of identity and evolutionary divergence among different species can be evaluated through the amino acid differences of SMN protein. *Homo sapiens*, *Mus musculus*, *Danio rerio*, *Xenopus laevis*, *Caenorhabditis elegans*, *Drosophila melanogaster*, and *Schizosaccharomyces pombe* have wide evolutionary distances, with range of SMN protein conservation from 83 to 18.9% identity. However, the N-terminal Gemin2 binding domain, the central Tudor domain, and the C-terminal YG box are highly homologous among all species (Osman et al., 2019). Among the invertebrate models, *C. elegans*, together with *D. melanogaster*, represents the most exploited animal model for High-throughput screening studies for *in vivo*, allowing to study the mechanisms of MN degeneration underlying SMA and to test compounds. Its small size, short life cycle, body transparency, ease to generate transgenic animals, and low maintenance costs contribute to its large use in experimental studies. Moreover its nervous and locomotor systems are well known, since its 302 neurons and 95 body wall muscles are all identified (C. elegans Sequencing Consortium, 1998; Dimitriadi and Hart, 2010; Wolozin et al., 2011; Cáceres et al., 2012; Lee et al., 2013; de Carlos Cáceres et al., 2018). To date, several *C. elegans Smn* mutants have been developed, such as smn-1(ok355) (null-mutant form) and smn-1(cb131). The first one bears a wide deletion of most *Smn* coding region, leading to growth and fertility defects, MN loss and to early death (Briese et al., 2009; Dimitriadi and Hart, 2010; Grice et al., 2011). On the other hand, smn-1(cb131) model shows a point mutation in N-terminal domain and, while displaying a similar MN degeneration to smn-1(ok355), it survives longer allowing screening progression (Grice et al., 2011; Sleigh et al., 2011). Neuromuscular and motor defects can be analyzed in the worm by the “thrashing assay,” a test to measure the number of lateral swimming movements (Buckingham and Sattelle, 2009). These evaluations can be also correlated to MN degeneration analysis. To this aim, the most recent automated system has been developed by de Carlos Cáceres group: it employs microfluidic and image analysis that assess worm phenotypes analyzing D-type ventral MN degeneration through a quick genetic screening technique (De Carlos Cáceres et al., 2018).

Another invertebrate animal, used for both DS and DR, is *Drosophila melanogaster*: it shares some advantages of *C. elegans*, as a rapid life cycle, an easy husbandry and a simple genetic manipulation (Cauchi and Van Den Heuvel, 2007; Lessing and Bonini, 2009; McGurk et al., 2015; Aquilina and Cauchi, 2018). Different *D. melanogaster Smn* mutants have been set up: smn73Ao (smn^A^) and smn^B^ mutants bear point mutations in C-terminal YG box (Chan et al., 2003; Chang et al., 2008; Grice et al., 2011); smnf05960 (smn^C^) and smnf01109 (smn^D^) mutants carry a transposon insertion respectively downstream and inside the Tudor domain. All these mutant models display impaired motor behavior, neuronal transmission and NMJ defects, leading to early death at larval moult stage (Grice et al., 2011). Finally, the EY14384 (smnE33) mutant fly carries a transposon insertion upstream of putative transcription start site and, while showing similar motor defects to the other mutants, it has a higher survival (Rajendra et al., 2007; Chang et al., 2008; Grice et al., 2011). However, the most recent fly SMA model has been developed by inserting the human *SMN2* minigene reporter, fused to luciferase, into the *Drosophila* genome, in order to easily obtain the exon 7-inclusion during *SMN2* splicing process. This cheap and feasible model allows to rapidly screen thousands of chemicals, possibly increasing FL-SMN protein levels (Konieczny and Artero, 2020).

*Danio rerio*, also known as zebrafish, is another useful invertebrate model to screen many compounds by DS/DR approaches. It is commonly used because of its high gene homology with human species and low maintenance costs (Howe et al., 2013; Patten et al., 2014). However, it has been mainly employed for ALS and HSP studies, whereas few SMA zebrafish models exist. One SMA model was developed by injecting an antisense morpholino oligonucleotide against *Smn* gene. Other zebrafish models bear the following gene mutations: smn^G264D^, smn^Y262X^ and smn^L265X^ and are all similarly characterized by muscle weakness and atrophy, with halved lifespan compared to WT controls (Boon et al., 2009; Patten et al., 2014). To further increase the fish survival, the human *SMN2* gene has been inserted in the genome of smn^Y262X^ fishes: such insertion increased SMN protein levels and slightly improved the animal survival (Hao et al., 2011).

Among the vertebrate models, *Mus musculus* is the most valuable one, especially for the Phenotypic screening. Murine and human genes display 90% of homology, even though mouse has just 20 chromosome pairs, while humans 23. Mouse genome has been widely manipulated to develop several neuromuscular disease models (transgenic, knockin, chimeras, and knockout), including SMA (Mouse Genome Sequencing Consortium et al., 2002; Fisher and Bannerman, 2019), taking into account that mice possess only one *Smn* gene and none *SMN2* gene copies (Monani, 1999). Some of the SMA murine models recapitulate the human severe disease form (type I), such as SMN^−^ and FVB.*SMN2*;*Smn* ^−^ (referred also as Burghes’ severe model incipient congenic); other ones (such as FVB.SMNΔ7 model) mimic the intermediate disease form (type II); milder forms (type III and IV) have been also developed [respectively, *Smn* A2G (also referred as Burghes type III model incipient congenic) and *Smn1* ^c^, and FVB.Cg-*Smn1*<tm1Msd> Tg (*SMN2*)566Ahmb/J] (Osborne et al., 2012; Edens et al., 2015; Eshraghi et al., 2016). Moreover there is also the “SMA-like” murine model, mainly referred as Taiwanese model: these animals, carrying the *Smn* ^−/−^ *SMN*2 genotype, are classified into the three pathological SMA form groups (type 1, 2 or 3) based on their phenotypes (Hsieh-Li et al., 2000).

The most used SMA murine model for DS/DR is certainly the SMNΔ7 mouse: it is a triple homozygote, characterized by selective spinal MN degeneration, progressive muscle atrophy, reduced body weight, early (from postnatal day 5, P5) motor performance impairment, and premature death (around P14) compared to WT littermates (Edens et al., 2015; Simon et al., 2017; Kannan et al., 2018; Rimer et al., 2019; Tejero et al., 2020). The other available SMA models are generally excluded from DS/DR studies, since they die too early (by P5, or even embryonic, respectively in the case of FVB.*SMN2*;*Smn* ^−^ and SMN^−^) or too late [*S*mn A2G and *Smn1* ^c^, and FVB. Cg-*Smn1*<tm1Msd> Tg (*SMN2*)566Ahmb/J], hence making difficult to evaluate the tested compound efficacy or requiring excessively extended observational studies.

## Pathways Targeted by Drug Screening and Drug Repositioning Approaches in Spinal Muscular Atrophy

As stated, only three drugs are currently authorized for administration to patients. To further increase the available therapeutic options, DS/DR approaches can be pivotal since they can identify new or repurposed effective drugs.

**FIGURE 2:**
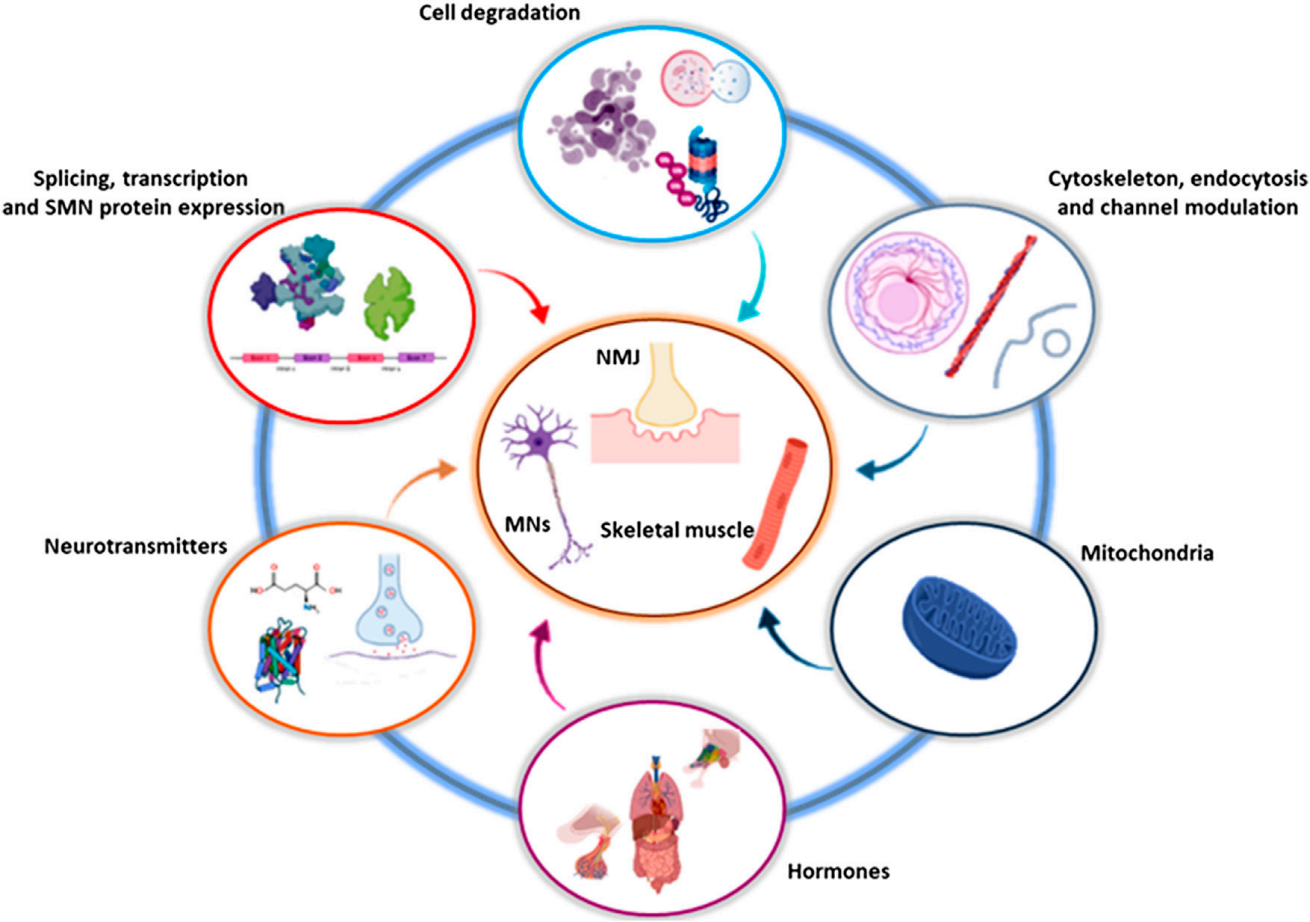
Involved and converging pathways targeted by DS and DR. The drugs identified by DS and DR influence and converge on a limited number of cellular and molecular pathways, that in turn act on specific districts, in particular involving MNs, NMJs and skeletal muscles. Created with BioRender software. DR, drug repositioning; DS, drug screening; MN, motor neuron; NMJ, neuromuscular junction; SMN, survival motor neuron.

Obviously, the attention is primarily focused on targeting pathways involved in *SMN*-specific transcription regulation. However, current expert opinions suggest not neglecting *SMN*-independent cascades and different cell targets, such as pathways involving degradation processes, cytoskeletal modulation, cell signaling and oxidative mechanisms (Figure 2). Below we describe natural, chemical and/or FDA-approved compounds, which have been discovered and tested through DS/DR studies, classifying them on the basis of their molecular mechanisms of action. The entirety of the described compounds are also resumed in Table 1.

**TABLE 1:** List of compounds discovered by DS or DR approaches for SMA treatment.

Molecular mechanism	Compound	Molecular class	DS/DR	Original target disease (for DR)	Clinical trial phase	Ref. And/or clinical trial
Direct and indirect modulation of *SMN*2 transcription	Branaplam (LMI070; NVS-101)	Pyridazine-derivative	DS	—	Active, not recruiting-phase II clinical trial for SMA	NCT02268552
	RG7800 (RO6885247)	Small molecule	DS	—	Stopped-phase I/II clinical trial for SMA	NCT02240355
	Risdiplam (RG7916; RO7034067)	Small molecule (RG7800 derivative)	DS	—	Currently in phase II clinical trial for SMA	NCT02913482 (Firefish), NCT02908685 (Sunfish), NCT03032172 (Jewelfish), NCT03779334 (Rainbowfish), NCT04256265, NCT04177134
	RG3039 (PF-06687859; D157495)	Quinazoline	DS	—	Suspended after phase I clinical trial for SMA	Gogliotti et al. (2013)
	Sodium vanadate	Inorganic sodium salt	DS	—	—	Liu et al. (2013)
	LDN-76070	Small molecule	DS	—	—	Cherry et al. (2013)
	LDN-75654	Small molecule	DS	—	—	Cherry et al. (2013)
	*Brucea javanica*	Simaroubaceae family’s medicinal plant	DS	—	—	Baek et al. (2019)
	Sodium butyrate	Organic sodium salt	DR	—	Recruiting-clinical trial for diabetes mellitus, type 1; several completed-trials (for shigellosis; gut health, SCFA metabolism, breast cancer, alcohol dependence, contact dermatitis, obesity)	Chang et al. (2001)
	Sodium phenylbutyrate	Sodium butyrate analogue	DR	Urea cycle disorder	FDA-approved	Andreassi et al. (2004)
					Completed-clinical trial in SMA	NCT00528268 (STOPSMA), NCT00439218 (NPTUNE02) and NCT00439569 (NPTUNE01)
	Valproic acid	Synthetic derivative of propylpentanoic acid	DR	Seizures; status epilepticus; bipolar disorder; migraine; schizophrenia	FDA-approved	Kissel et al. (2011)
					Completed-clinical trial in SMA	NCT00481013, NCT00374075
	Trichostatin A (TSA)	Natural derivative of dienohydroxamic acid	DR	Mycosis	Recruiting-clinical trials for tumors, seizures, osteoarthritis, anemia, infertility, ischemic stroke, opioid dependence	Avila et al. (2007)
					Many completed-trials including infectious diseases	
	Vorinostat (suberoylanilide hydroxamic acid; SAHA)	Synthetic hydroxamic acid derivative	DS/DR	Cutaneous T-cell lymphoma	FDA-approved	Hahnen et al. (2006)
	Panobinostat (LHB589)	Cinnamic hydroxamic acid analogue	DR	Multiple myeloma	FDA-approved	Garbes et al. (2009)
	Celecoxib	Cyclo-oxygenase 2 inhibitor	DR	Osteoarthritis, rheumatoid arthritis in adults; juvenile arthritis; ankylosing spondylitis, colorectal polyps; pain; dysmenorrhea; cardiovascular risk reduction	FDA-approved	NCT02876094
					Recruiting-phase II clinical trial for SMA	
	Hydroxyurea (Hydroxycarbamide)	Ribonucleoside diphosphate reductase inhibitor	DR	Chronic myelogenous leukemia; polycythemia vera; cervical, head, neck and ovarian cancers; melanoma; sickle cell anemia	FDA-approved	Grzeschik et al. (2005)
					Three completed-clinical trial for SMA	NCT00485511, NCT00568698, NCT00568802
	Aclarubicin	Oligosaccharide anthracycline antineoplastic antibiotic	DS/DR	Acute myeloid leukemia	FDA-approved	Andreassi et al. (2001)
	Moxifloxacin	Synthetic fluoroquinolone antibiotic	DS/DR	Respiratory tract, skin and skin structure, intra-abdominal and GI infections, endocarditis, tuberculosis, nongonococcal urethritis, plague, meningitis and other CNS infections	FDA-approved	Konieczny and Artero (2020)
	Rigosertib	Synthetic benzyl styryl sulfone analogue	DS/DR	Chronic myelomonocytic leukemia	Currently in phase III clinical trial for chronic myelomonocytic leukemia	Son et al. (2019)
						NCT02562443
	Indoprofene (K4277)	Cyclooxygenase (COX) inhibitor	DS/DR	—	Recalled from the market	Kim et al. (2020)
Cell death and degradation pathways	Levetiracetam (LEV; (S)-α-ethyl-2-oxo-pyrrolidine acetamide)	Pyrrolidine	DR	Seizures	FDA-approved	Ando et al. (2019)
						NCT00324454
	Liuwei dihuang extract (LWDH)	Chinese herbal formula	DR	Kidneys, liver and asthma; geriatric diseases	—	Tseng et al. (2017)
	Bortezomib	Dipeptide boronic acid analogue	DR	Multiple myeloma	FDA-approved	Foran et al. (2016)
	Z-Phe-Ala fluoromethyle ketone (Z-FA-FMK)	Cysteine proteases irreversible inhibitor	DS	—	—	Wang et al. (2019)
	E64d	Cysteine protease inhibitor	DS	—	—	Wang et al. (2019)
	Edaravone	Pyrazolone	DR	Amyotrophic lateral sclerosis	FDA-approved	Sun et al. (2019)
	L-carnitine	Amino acid derivative	DR	Primary and secondary carnitine deficiency; end-stage renal disease	FDA-approved	Kissel et al. (2011)
					Completed phase II clinical trial for combinatorial treatment with valproic acid in SMA	NCT00227266, NCT00661453
Mitochondria-related pathways	Olesoxime (TRO19622)	Cholesterol-like structure	DR	Amyotrophic lateral sclerosis	Completed phase III clinical trial for ALS	Bordet et al. (2007)
					Active, not recruiting-phase II clinical trial for SMA	NCT01302600, NCT02628743
Cytoskeleton dynamics, endocytic pathway and channel modulators	SRK-015	Monoclonal antibody	—	—	Active, not recruiting-phase II clinical trial for SMA	NCT03921528
	Fasudil	Heterocyclic aromatic organic compound	DR	Cerebral vasospasm; cerebral ischemic symptoms	PMDA approved in Japan	Bowerman et al. (2012)
					Recruiting-phase II clinical trial for ALS	NCT03792490, eudra-CT-nr.: 2017-003676-31
	Y-27632	Rock inhibitor	—	—	—	Hensel et al. (2017)
	Fampiridine (Fampyra; 4 aminopyridine)	Aromatic amine	DS/DR	Multiple sclerosis	FDA-approved	Sleigh et al. (2011)
					Completed-phase III clinical trial in SMA	
	Reldesemtiv (CY 5021; CK-2127107)	Small molecule	DR	Chronic obstructive pulmonary disease; amyotrophic lateral sclerosis	Currently in phase II clinical trial for COPD; ALS and SMA	Ramdas and Servais (2020)
						NCT02644668
Hormones signaling pathways	Somatotropin (growth hormone)	Hormone	DR	Chronic renal failure; turner and prader-Willi syndromes; growth disorders	FDA-approved pilot study for SMA	NCT00533221, NCT01369901
	Protirelin	Thyrotropin releasing hormone analogue	DR	Epilepsy; spinal cord injury; spinocerebellar ataxia; neonatal respiratory distress	FDA-approved	Tzeng et al. (2000)
	Taltirelin hydrate (TA-0910)	Thyrotropin releasing hormone analogue	DR	Spinocerebellar degeneration disease	Recruiting-phase IV clinical trial for SDD	Ohuchi et al., (2016)
						NCT0410774
	Prednisolone	Synthetic glucocorticoid	DR	Adrenocortical insufficiency; adrenogenital syndrome; hypercalcemia; thyroiditis; rheumatic, ocular, oral, hematologic disorders; collagen, dermatologic, lung, gastrointestinal, neoplastic and liver diseases; asthma; pericarditis; multiple sclerosis; myasthenia gravis¸ organ transplants; nephrotic syndrome	FDA-approved	Quattrocelli et al. (2017)
Neurotransmitters' modulation	Riluzole	Benzothiazole derivative	DR	Amyotrophic lateral sclerosis	FDA-approved	Dimitriadi et al. (2013)
					Completed phase III clinical trial for SMA	NCT00774423 (ASIRI)
	Ceftriaxone	Third generation cephalosporin antibiotic	DR	Acute otitis media; endocarditis; meningitis; septicemia; antibiotic prophylaxis; bone, joint, gastrointestinal, intra-abdominal, respiratory tract, skin and urinary tract infection	FDA-approved	Nizzardo et al. (2011)
	Lamotrigine	Synthetic phenyltriazine	DR	Lennox–Gastaut syndrome; bipolar disorder and mood episodes	FDA-approved	Nascimento et al. (2010)
	Gaboxadol hydrochloride	Synthetic compound	DS	—	—	Sleigh et al. (2011)
	Gabapentin	GABA chemical analogue	DR	Postherpetic neuralgia; partial-onset seizures; peripheral neuropathic pain; painful diabetic neuropathy	FDA-approved	Merlini et al. (2003)
					Two clinical trials completed on SMA type II and III patients	
Neuromuscular junction stabilization	Amifampridine (pyridine-3,4-diamine, 3,4-diaminopyridine, 3,4-DAP)	Organic compound pyridine-derived	DR	Lambert-Eaton myasthenic syndrome (LEMS)	FDA-approved	NCT03781479, NCT03819660
					Recruiting-phase II for SMA	
	Tideglusib (NP-12, NP031112)	Small heterocyclic thiadiazolidine-based molecule	DS/DR	Alzheimer’s disease; progressive supranuclear palsy; congenital myotonic dystrophy	Completed phase II clinical trial for Alzheimer’s disease;	Makhortova et al. (2011)
					Not yet recruiting phase III clinical trial for congenital myotonic dystrophy	NCT03692312
	Salbutamol (albuterol)	Selective beta2-adrenergic receptor agonist	DR	Asthma, chronic obstructive pulmonary disease	FDA-approved	Frongia et al. (2019)
					Recruiting-clinical trial for SMA in French register	NCT04177134

DR, drug repositioning; DS, drug screening; FDA, Food and Drug Administration; SMA, spinal muscular atrophy. Natural, chemical and FDA-approved compounds are classified by their mechanism of action. The SMA clinical study phases and the relative trial identifiers (NTC number from ClinicalTrials.gov) are indicated; when lacking, clinical trials referring to other pathologies, together with the most recent references to experimental SMA studies, are shown.

### Direct and Indirect Modulation of Survival Motor Neuron 2 Transcription

During the last years, many studies are aiming to identify new *SMN*-dependent approaches, more effective and less invasive than the available ones.

*SMN2* pre-mRNA splicing modulators (as Nusinersen) can significantly improve child health status. In this context, Branaplam is the first small compound orally administered and it is currently in phase II clinical trial (involving SMA type 1 patients; ClinicalTrials.gov identifier NCT02268552). It was developed by improving effectiveness, bioavailability and safety of pyridinazine, discovered as hit compound by High-throughput screening in NSC34 cell line with *SMN2* mini-gene reporter inclusion (Palacino et al., 2015; Cheung et al., 2018). In detail, it stabilizes U1 snRNP binding with 5′ splice site, leading to exon seven inclusion in *SMN2* mRNA, thus increasing *SMN2*-derived FL-SMN level (Palacino et al., 2015; Shorrock et al., 2018). Another small molecule, known as RG7800, was discovered using a similar screening. PTC Therapeutics, in collaboration with Roche, identified RG7800, then tested in the MOONFISH clinical trial. However, the trial was preventively stopped between phase I and II because of a RG7800-dependent eye toxicity observed in a long-term concomitant treatment study performed in monkeys (Identifier: NCT02240355) (Naryshkin et al., 2014; Kletzl et al., 2019). Therefore, RG7800 was optimized developing a new oral drug, RG7916, known as Risdiplam, which was tested in different cell lines and animal models (mice, rats, and monkeys) (Poirier et al., 2018; Ratni et al., 2018). Risdiplam is currently under evaluation in the four clinical studies mentioned above in *Spinal Muscular Atrophy Approved Drugs* (Kletzl et al., 2019; Haniff et al., 2020).

From the beginning of 21st century until now, other synthetic, inorganic and natural compounds were discovered via DS approaches, acting as splicing modifiers and up-regulators of *SMN2*-derived FL-SMN. Among them, the 2,4-diaminoquinazoline class was found by Jarecki and coll., after the remarkable screening of 550,000 synthetic compounds using NSC-34 cell line containing the *SMN2* promoter β-lactamase reporter gene (Jarecki et al., 2005). The quinazoline analog compound RG3039 is a DcpS inhibitor that assured an overall improvement of disease phenotype when tested on Taiwanese and 2B/− and on SMA2B/− mice (Gogliotti et al., 2013). Based on this experimental evidence, Repligen conducted a clinical trial which initially provided successful results, but, following the take-over by Pfizer, the phase I trial was suspended on June 2014, because of a limited SMN increase in SMA children blood (Pfizer Pulls Plug on Repligen SMA Collaboration, 2015).

In addition, *SMN2* modulators such as Sodium vanadate, small molecules (LDN-76070 and LDN-75654) and Brucea Javanica extract were originally identified via cell-signaling assays, but they gave insufficient results to be tested in SMA mice (Zhang et al., 2001; Cherry et al., 2013; Baek et al., 2019). In particular, Sodium vanadate showed to be effective in Taiwanese type III SMA mice when administered in combination with the detoxification agent L-ascorbic acid: however, their effects remain to be confirmed in more severe mouse models of SMA (Zhang et al., 2001; Liu et al., 2013; Seo et al., 2013). While Brucea Javanica has been recently tested on SMAΔ7 mice and the available preliminary results still require more detailed studies, LDN-76070 and LDN-75654 administration in the same mouse model increased lifespan, motor functions and SMN protein levels (Cherry et al., 2013; Baek et al., 2019).

In addition, histone deacetylase (HDAC) inhibitors, revealed by DR studies, could be promising also in case of SMA. Several FDA-approved HDAC inhibitors (sodium phenylbutyrate, valproic acid, Vorinostat, trichostatin A, and Panobinostat) or not yet approved (sodium butyrate) have been investigated for SMA treatment (Chang et al., 2001; Sumner et al., 2003; Andreassi et al., 2004; Hahnen et al., 2006; Avila et al., 2007; Garbes et al., 2009). In addition to promoting *SMN2* transcription, they can affect the expression of many other genes (Calder et al., 2016; Poletti and Fischbeck, 2020). In combination with Nusinersen, HDAC inhibitors exerted synergistic effects, further enhancing the expression of *SMN2* in human SMA fibroblasts (Pagliarini et al., 2020). Importantly, this combinatorial strategy could lead patient benefits, hypothesizing lower or less frequent Nusinersen doses, and consequently reducing repeated intrathecal administrations and high costs (Poletti and Fischbeck, 2020; Ramdas and Servais, 2020).

Other FDA-approved drugs arisen from DR studies and able to modulate *SMN2* are celecoxib and Hydroxyurea; they are both important enzymatic inhibitors. Celecoxib is a non-steroidal anti-inflammatory cyclo-oxygenase two inhibitor, mainly used to treat rheumatoid arthritis and osteoarthritis (Seibert et al., 1994; Lipsky and Isakson, 1997). Its potential role in SMA therapy was deepened by Farooq’s lab, which focused on p38 MAPK pathway and showed that it upregulates cytoplasmic HuR protein, in turn able to stabilize mRNA-binding, also involving *SMN* (Farooq et al., 2013). Celecoxib, tested on Human neuron-committed teratocarcinoma (NT2) and mouse motor neuron-derived (MN-1) cell lines and on SMAΔ7 mice, induced the *SMN2*-derived FL-SMN mRNA stabilization. In addition, since it crosses the BBB, celecoxib is an optimal candidate for SMA therapy; indeed, it is currently in phase II clinical trial (Identifier: NCT02876094 (Farooq et al., 2013; Wadman et al., 2020). Instead, Hydroxyurea, a ribonucleoside diphosphate reductase inhibitor, prevents the exit from cell cycle G1/S phase and promotes fetal hemoglobin production. For these reasons, it is used to treat many neoplasias such as melanoma, chronic myelogenous leukemia, polycythemia vera, cervical and ovarian cancers, head and neck tumors, and sickle cell anemia (Rodgers et al., 1990; Madaan et al., 2012). Due to its gene interaction ability, Hydroxyurea was evaluated as therapeutic candidate for SMA, showing excellent results in lymphoblastoid cell lines isolated from type I, II, and III SMA patients (Grzeschik et al., 2005). However, three clinical trials (Identifiers: NCT00568698, NCT00568802, and NCT00485511) did not confirm its efficacy in improving motor functions of SMA types II or III (Wadman et al., 2020).

Although DS and DR are often carried out separately, their combination allowed to discover unusual drugs for neurodegenerative disease treatment. For example, Aclarubicin, an oligosaccharide anthracycline antineoplastic antibiotic, used in case of Acute Myeloid Leukemia treatment (Mitrou, 1990), was identified in 2001 via High-throughput screening on SMA type I fibroblast cell line and NSC-34 cell line containing *SMN2* minigene reporter: Aclarubicin increases *SMN2* exon seven inclusion, upregulating FL-SMN expression. Indeed, Aclarubicin seems to act as a transcriptional activator, inducing by indirect pathways *SMN2* exon seven inclusion (Morceau et al., 1996; Andreassi et al., 2001). Likewise, Moxifloxacin, a synthetic fluoroquinolone antibiotic used to treat several infections, was identified by performing High-throughput screening study in a *Drosophila SMN2* minigene reporter model, as described above (Konieczny and Artero, 2020). Moxifloxacin modulates *SMN2* splicing by promoting exon seven inclusion and crosses the *Drosophila* BBB; moreover, it increases FL-SMN expression in HeLa cell lines. The authors showed that Moxifloxacin exerts a dose-dependent increase of Serine/arginine Rich Splicing Factor 1 (SRSF1) levels promoting the *SMN2* exon seven inclusion (Konieczny and Artero, 2020).

Finally, Rigosertib and Indoprofene have been also proposed for SMA therapy, after DS identification. Rigosertib is a synthetic benzyl styryl sulfone analogue currently in phase III clinical trial for chronic myelomonocytic leukemia care (Garcia-Manero et al., 2016); it acts as a *SMN2* splicing modifier, as suggested by a screening of small molecules carried out on C33A cell lines containing a *SMN2*-luciferase minigene reporter and SMA type I fibroblast cell lines (Son et al., 2019). Similarly, Indoprofene is a cyclooxygenase (COX) inhibitor, used as analgesic and anti-inflammatory drug (Paeile et al., 1989): it can increase SMN levels both in *SMN2*-luciferase cells and in type I SMA patient fibroblasts, and enhance the viability of a transgenic Type I SMA mouse model (Monani, 2000). Moreover, while showing also positive effects mediated by PDK1/AKT pathway on muscle wasting as demonstrated in aged mice (Kim et al., 2020), up to now it was never tested in clinical trials for SMA.

### Cell Death and Degradation Pathways

The above-mentioned approaches are merely *SMN*-dependent strategies, but there are numerous studies suggesting that further cellular mechanisms can affect the severity of SMA. Indeed, DS and DR approaches represent a valid strategy to identify molecules also acting on *SMN*-independent pathways.

Recent studies suggest that the lack of SMN increases the cleavage of caspase-3, and triggers the apoptotic pathway and MN degeneration (Piras et al., 2017; Maretina et al., 2018; Schellino et al., 2018). In this context, the repositioning of the anti-epileptic drug Levetiracetam (trade name Keppra) decreased the cleaved-caspase three expression in SMA-iPSCs-MNs (Ando et al., 2019). Likewise, Bax and Bcl-xL proteins, respectively mediating anti- and pro-apoptotic pathways, seem to be influenced by SMN lack, with consequent downstream effects. The expression levels of Bax are significantly increased in the spinal cord of SMA mice, and the overexpression of Bcl-xL increases SMN-reduced MN survival (Garcera et al., 2011) and can extend SMA mice lifespan (Tsai et al., 2006; Tsai et al., 2008). Moreover, a decrease in the levels of Bcl2 in postmortem spinal cord from SMA I fetuses and SMAΔ7 mice was reported (Soler-Botija et al., 2003; Tsai et al., 2008; Piras et al., 2017). Based on this evidence, some authors proposed the repositioning of the water extract of Liuwei dihuang (LWDH-WE): despite not being FDA approved, it is a herbal formula widely used in the traditional Chinese medicine as treatment for kidney and liver disorders, asthma and geriatric diseases (Sangha et al., 2012; Tseng et al., 2014). When tested on Parkinson’s disease (PD) mouse models, it improved their motor activity, suggesting its use also for SMA treatment (Tseng et al., 2014; Tseng et al., 2017). Indeed, LWDH-WE was able to attenuate SMN deficiency-induced down-regulation of Bcl-2 and decreased cytosolic cytochrome c and cleaved caspase-3; the drug also counteracted cell death in NSC34 cells thanks to its SMN-promoting and antiapoptotic activities (Tseng et al., 2017).

Moreover, the ubiquitin/proteasome system is also altered in SMA, due to SMN lack (Powis et al., 2016). DS-based comparative proteomics revealed a significantly decrease of ubiquitin-like modifier activating enzyme 1 (UBA1) level in SMA mice. Since SMN directly influences UBA1 level, and its overexpression in SMA mice improves motor functions and increases their survival (Wishart et al., 2014; Powis et al., 2016), several works suggest to pharmacologically modulate its levels to influence SMA progression (Patten et al., 2014; Maretina et al., 2018). On the other hand, by using repositioning strategy involving the use of proteasome inhibitors, it was showed that the chemotherapy drug Bortezomib blocks SMN degradation in peripheral tissues and improves motor functions in SMAΔ7 mice (Kwon et al., 2011; Foran et al., 2016).

Other degradation pathways have been recently investigated. Wang and collaborators tested different drugs from a small library and demonstrated the involvement of both non-lysosomal (calpain 1/2) and lysosomal cysteine proteases (CTSL/CTSB) in degrading SMN proteins (Wang et al., 2019). In particular, establishing a versatile *SMN2*-GFP reporter cell line, they identified a novel role of the cysteine protease inhibitor Z-FA-FMK: this compound increased the level of functional SMN by inhibiting the protease-mediated degradation of both FL-SMN and delta7 SMN. Z-FA-FMK and the analogous compound E64days, previously used as Alzheimer’s disease (AD) and brain injury treatments, rescued MN degeneration in SMA, suggesting that inhibiting protease-mediated degeneration could be a potential therapeutic for SMA (Wang et al., 2019).

Finally, the repositioning of Edaravone and the Acetyl-L-carnitine (both drugs effective in oxidative stress modulation) has been also evaluated. Edaravone (3-methyl-1-phenyl-2-pyrazolin-5-one), a free radical scavenger, showed efficacy in acute brain infarction and in ALS, and worldwide it is now approved for the treatment of both these pathologies in several countries (Jackson et al., 2019; Sun et al., 2019). Edaravone reversed oxidative stress-induced apoptosis and inhibited mitochondrial reactive oxygen species upregulation in SMA-iPSCs-derived spinal MNs (Ando et al., 2017). Instead, the L-carnitine is an anti-oxidant natural compound involved in cellular lipid peroxidation and known to inhibit mitochondrial damage and mitochondrial-mediated apoptosis both *in vitro* and *in vivo* (Bigini et al., 2002). Its exogenous administration in the acetylated form (Acetyl-L-carnitine, ALC), alone (Merlini et al., 2002, 2010; Wadman et al., 2020) or in combination with the valproic acid (Swoboda et al., 2010; Kissel et al., 2011; Wadman et al., 2020), was tested in two different SMA trials. However, on one hand, although the administration of ALC alone seemed effective, it did not allow to draw a final conclusion due to a too small cohort of SMA patients (Merlini et al., 2010); on the other hand, the combinatorial treatment of ALC with valproic acid compared to placebo did not reach significant improvements of motor function and muscle strength (Swoboda et al., 2010; Kissel et al., 2011; Wadman et al., 2020).

### Mitochondria-Related Pathways

Mitochondria are organelles highly impacted at the very early stage of many neurodegenerative diseases, to the point to be considered as a possible unifying trait in the pathogenesis of these disorders (for an extended review see Stanga et al., 2020). Mitochondrial dysfunctions are reported as important pathological mechanisms in MN disorders (Patten et al., 2014). In SMA, mitochondrial impairment occurs in many tissues, both at the level of central and peripheral nervous system. This is probably due to the fact that i) mitochondria are particularly present in axons of neuronal cells, heart cells and skeletal muscles, and ii) *SMN* is ubiquitously expressed in the body. Indeed, SMN deficiency has been correlated to oxidative stress, mitochondrial dysfunction and deregulation of bioenergetic pathways. Therefore, treatments targeting mitochondria could represent a new promising solution not only for SMA, but also for many other disorders (Panuzzo et al., 2020).

Also in the case of mitochondria-related pathways, DR and DS approaches helped in the identification of promising drugs targeting mitochondria in SMA. Drugs targeting mitochondrial proteins and channels, such as the Na^+^/Ca^2+^exchanger (Annunziato et al., 2020), could be promising in the SMA treatment: the modulation of NCX2 (sodium calcium exchanger isoform 2) expression, by microRNA-206 administration in SMAΔ7 mice, delayed the disease progression and improved behavioral performance in mice (Valsecchi et al., 2020).

Another example is Olesoxime, originally evaluated for diabetes since able to promote the survival of the pancreatic β-cells, which are particularly rich in mitochondria. Interestingly, DR studies revealed important positive effects also for SMA. Indeed, Olesoxime is able to bind proteins of the outer mitochondrial membrane: there, it reduces its permeability when exposed to stress (Pruss et al., 2010) preventing, in turn, apoptosis by reducing release of pro-apoptotic factors and maintaining energy production (Kariyawasam et al., 2018). In this way, Olesoxime can preserve the integrity of MNs (Bordet et al., 2007). Moreover, Olesoxime showed neuroprotective and neuroregenerative effects in several animal models of motor nerve degeneration and in a transgenic mouse model of severe SMA (SmnF7/F7; NSE-Cre mice; mutant mice carrying a deletion of *Smn* exon seven directed to neurons): daily administrations of Olesoxime extended the survival of the treated mice (Bordet et al., 2010). Taken together, these data suggest that Olesoxime might maintain MN function and might be a therapeutic drug in the treatment of SMA. However, unfortunately, oral administration of Olesoxime in phase II trial for SMA (and phase III for ALS) failed, because it did not show sufficient efficacy (Bordet et al., 2007; Swalley, 2020).

### Cytoskeleton Dynamics, Endocytic Pathway and Channel Modulators

The exploitation of modern DS and DR approaches also allowed to identify new treatments aimed at improving or maintaining integrity and functionality of SMA axons/NMJs. In this context, perturbations of cytoskeleton dynamics, known to impair SMA MN neurogenesis, have been studied (Bowerman et al., 2007); therefore, the overexpression or inhibition of cytoskeletal remodeling modulators represent intriguing therapeutic strategies.

One of the main actin dynamics regulator is the RhoA-ROCK pathway. ROCK is a serine-threonine kinases and a downstream effector of the RhoA small GTPase. The RhoA/ROCK pathway is mainly involved in shape regulation and neuronal cells movement (extension and branching), by acting on the cytoskeleton dynamics (growth, differentiation, and retraction) and critically influencing MN synapse functions. An aberrant upregulation of RhoA/ROCK pathway was observed in SMA neuronal cell models and in SMA patients fibroblasts (Bowerman et al., 2007; Bowerman et al., 2010; Nölle et al., 2011; Koch et al., 2018). Moreover, the profilin IIa upregulation (due to SMN-deficiency) causes an upstream dysregulation of RhoA/ROCK pathway (Maretina et al., 2018). This evidence suggests that ROCK inhibition can induce beneficial effects in SMA, as already demonstrated for several neurodegenerative diseases (Hensel et al., 2015), including SMA: indeed, Y-27632 and Fasudil are two RhoA/ROCK inhibitors, able to extend lifespan and improve motor functions in Taiwanese and *SMN2*B/− SMA mice (Bowerman et al., 2010; Bowerman et al., 2012; Bowerman, 2014; Hensel et al., 2017).

Hensel tested the combination of Y-27632 with an ERK pathway inhibitor, using an automated MN cell-bodies High-throughput detection screening on primary spinal cord cultures. The simultaneous inhibition of both pathways induced synergistic beneficial effects, significantly increasing MN viability, with respect to the single inhibition of one of them (Hensel et al., 2017).

Fasudil is a vasodilator drug, used for the treatment of cerebral vasospasm and delayed cerebral ischemic symptoms after aneurysmal subarachnoid hemorrhage. In Japan its use has been approved by the Pharmaceuticals and Medical Devices Agency since 1995 (Shibuya and Suzuki, 1993; Zhao et al., 2006) and is currently tested in clinical studies for disorders such as the Raynaud phenomenon, atherosclerosis and ALS (ROCK-ALS trial, NCT03792490, Eudra-CT-Nr.: 2017-003676-31). Interestingly, Fasudil improved survival and promoted skeletal muscle development in SMA *Smn2*B/− mice, restoring the correct function of actin in MNs and supporting the formation of functional NMJs (Bowerman et al., 2012; Koch et al., 2018). Therefore, while Y-27632 is mainly used as an experimental biochemical tool in the study of ROCK signaling pathways, the ongoing clinical study of Fasudil for ALS patients could pave the way for the therapeutic evaluation of ROCK inhibitors in MN diseases by strengthening its DR in SMA field (Bowerman et al., 2017).

In addition, several SMA modifiers have been proposed as SMA therapy in combination with *SMN*-enhancing treatments. In particular, the contribution to SMA pathogenesis of Plastin 3 (PLS3) and Neurocalcin Delta (NCALD) proteins has been deeply evaluated. PLS3 is a Ca^2+^-dependent actin-binding protein, important for axonogenesis by increasing F-actin levels, and acting as positive regulator of endocytosis process. PLS3 is a powerful modifier of SMA: high levels of PLS3 have been reported in unaffected subjects carrying *SMN1* mutations; moreover PLS3 overexpression increased cell survival, supported neurite overgrowth and restored impaired endocytosis in *vitro* and *in vivo* SMA models (Oprea et al., 2008; Hosseinibarkooie et al., 2016a; Alrafiah et al., 2018; Maretina et al., 2018). PLS3 overexpression, combined with the subcutaneous injection of ASOs, has been recently confirmed to improve the survival of SMA mice, motor functions and NMJ size. Since their beneficial synergistic effects were greater than those obtained with ASO administration alone, further DS studies are recommended to better define effective therapeutic combinatorial strategies (Hosseinibarkooie et al., 2016b; Kaifer et al., 2017).

Analogous positive results have been obtained by combining NCALD and ASO treatment (Riessland et al., 2017). Riessland’ group identified NCALD as a potential SMA modifier by Genome-Wide Linkage and Transcriptome-Wide differential expression analysis performed on samples of SMA type 1 patients and fully asymptomatic people, both carrying homozygous *SMN1* deletions (Riessland et al., 2017). Furthermore, this work demonstrated the role of NCALD as negative regulator of endocytosis, since its knockdown effectively ameliorated these dysfunctions, supporting motor axon development and improving MN circuitry and NMJ presynaptic function in SMA models (worm, zebrafish, and mice) (Riessland et al., 2017). Given this evidence, DS and DR approaches confirmed their important contribution in revealing potential SMA disease modifiers involved in the modulation of different signaling pathways.

Another important example comes from Sleigh’s group, that in 2011, using a SMA type III *C. elegans* model, screened 1040 FDA-approved compounds of the NINDS library, to identify effective drugs targeting nerve/muscle activity (Sleigh et al., 2011). The results of this analysis suggested 4-aminopyridine (4-AP) as a candidate drug for SMA treatment: it is a dose-dependent potassium channel blocker, able to restore demyelinated neuron conductance and enhance synaptic transmission (by increasing pre-synaptic calcium influx into neurons). Administration of 4-AP rescued mutant *C. elegans* motility (Sleigh et al., 2011). Interestingly, 4-AP is the active ingredient of Fampiridine drug, also named Ampyra or Fampyra respectively in United States and Europe, and approved by FDA and EMA in 2011. It is already administered to multiple sclerosis and Lambert-Eaton myasthenic syndrome patients; but it has been also tested for spinal cord injury and PD (Jensen et al., 2011; Pérez Luque et al., 2015; Acorda Therapeutics Inc., 2017).

Therapeutic strategy of drug targeting signaling pathways involving Ca^2+^ influx modulation has been developed specifically to enhance muscle functions, as suggested by Citokinetics-Astellas ongoing trial of Reseldemtiv. Reldesemtiv, previously known as CY 5021 and CK-2127107, is a selective small-molecule fast skeletal muscle troponin activator, to date in trials for chronic obstructive pulmonary disease and ALS treatment (Shefner et al., 2018; Rossiter et al., 2019). It improves muscle contractility by increasing the affinity between troponin C and Ca^2+^, providing a therapeutic target for several skeletal muscle-related diseases, including SMA. Indeed, FDA attributed to Reldesemtiv the orphan drug designation as potential SMA therapeutic (Orphan designation: Reldesemtiv, Treatment of SMA, 2019). Its efficacy and tolerability in increasing muscle contraction force was demonstrated respectively in preclinical studies and phase one SMA trials (Calder et al., 2016). The following phase 2 trial (Identifier: NCT02644668) confirmed Reldesemtiv efficacy, when orally administered to patients with SMA types II, III, and IV, without observing dose-limiting safety or tolerability issues. A confirmatory phase 3 study is planned in the next years (Rudnicki et al., 2018; Ramdas and Servais, 2020).

### Hormone Signaling Pathways

The negative effects of SMN-deficiency, although mainly involved in MN impairment, also extend, as multisystem pathologies, to other components of the motor circuits and modulators of skeletal muscle development and function (Zhou et al., 2016). Among the others, the pharmacological modulation of various hormones and their signaling pathways is being studied in order to minimize muscle atrophy and injury by acting on neuronal regeneration, peripheral reinnervation and muscle growth (Tuffaha et al., 2016; Lopez et al., 2019). In addition, these drugs, mostly already approved by the FDA, could quickly be suitable for clinical translation.

For example, the administration of hypothalamic and pituitary hormones [growth hormone (GH) and thyrotropin releasing hormone (TRH)] or synthetic glucocorticoids (e.g., prednisolone), administered in case of several neuromuscular diseases can exert beneficial effects on muscle functions (Wadman et al., 2020). Indeed, GH induces insulin-like growth factor-1 (IGF-1) secretion at muscle and liver level: then, IGF-1 stimulates the physiological growth of long bones and soft tissues and muscle development, whereas in case of trauma, it supports muscle regeneration by promoting myogenic differentiation and myocyte hypertrophy (Tritos and Klibanski, 2016). GH administration is already clinically employed in chronic renal failure, Turner and Prader-Willi syndromes, growth disorders; furthermore, it has been studied for its beneficial effects in enhancing nerve regeneration and muscle reinnervation, following peripheral nerve injuries (Tuffaha et al., 2016; Lopez et al., 2019). Therefore, a DR for these natural or synthetic hormones can be suggested for SMA treatment.

Indeed, several studies have shown that intracerebral injections of IGF-1 in SMA mice supported survival and improved motor functions, preventing muscle atrophy and preserving NMJs (Bosch-Marcé et al., 2011; Shababi et al., 2011; Tsai, 2012; Tsai et al., 2014; Wadman et al., 2020); however, similar results have not yet been obtained in SMA patients (Kirschner et al., 2014).

Beneficial effects of TRH tripeptide, Glu-His-Pro-NH2, have been also observed in SMA skeletal muscles (Wadman et al., 2020). The TRH, in addition to stimulate the release of thyroid-stimulating hormone, seems involved in neuronal activity by its association with serotonin (Tzeng et al., 2000). Its synthetic analogs, i.e., Protirelin and Taltirelin hydrate, have been used to treat epilepsy, spinal cord injury, spinocerebellar ataxia, and neonatal respiratory distress (Shimizu et al., 1989; Tzeng et al., 2000; Urayama et al., 2002). Since TRH has been found in the spinal MNs, its role in the pathogenesis of MN diseases has been suggested, even if a TRH-based trial in ALS was unsuccessful (Brooks et al., 1987). However, its beneficial effects on motor functions and electromyographic results of SMA type II/III patients have been reported in different studies (small groups of infants with SMA type I in children with types II and III) and in a clinical trial (Takeuchi et al., 1994; Tzeng et al., 2000; Wadman et al., 2020).

Also the repositioning of a synthetic glucocorticoid drug prednisolone, currently used for Duchenne muscular dystrophy treatment, has been recently suggested for SMA therapy (Hoolachan et al., 2019). Prednisolone is also employed for the treatment of a wide spectrum of inflammatory conditions, involving allergies, autoimmune disorders and cancers (Gambertoglio et al., 1980; Frey, 1987). The beneficial effects of intermittent dosage of prednisolone in recovering skeletal muscles from injury, promoting sarcolemmal repair and limiting atrophic remodeling have been showed in the treatment of Duchenne muscular dystrophy patients and also in other neuromuscular diseases models (such as acute muscle injuries and muscular dystrophy mouse models) (Beenakker et al., 2005; Matthews et al., 2016; Quattrocelli et al., 2017). Moreover, prednisolone promotes the expression of Klf15, a transcription factor involved in muscle homeostasis and deregulated in pre-symptomatic SMA mice: this can further justify the positive effects, observed in SMA mice, including the improvement of muscle trophism and functioning and lifespan extension. Thus, this suggests that further investigations on the possibility of prednisolone repositioning as SMA therapy (Walter et al., 2018; Hoolachan et al., 2019).

Nevertheless, the studies were not completely free of bias, and further evaluations are required, in particular regarding TRH (Wadman et al., 2020). However, SMA-specific DS and DR studies concerning such hormone-based therapies still seem to be promising, as suggested by different works on SMA models reporting the benefits of their administration and recommending further related screenings (Kato et al., 2009; Ohuchi et al., 2016; Wadman et al., 2020).

### Neurotransmitters’ Modulation

Impairment of synaptic transmission has been also widely reported in SMA, suggesting that the pharmacological modulation of synaptic plasticity mechanisms could represent another therapeutic target and sustain the survival of MNs.

Different studies aimed at characterizing alterations of neurotransmission and abnormalities in SMA spinal circuitries both in *vitro* and *in vivo* models revealed hyperexcitability and loss of afferent proprioceptive synapses on MNs (Mentis et al., 2011; Gogliotti et al., 2012; Zhou et al., 2016; Bowerman et al., 2018), suggesting an impairment of glutamatergic synaptic transmission (Bowerman et al., 2018; Sun and Harrington, 2019). Impaired glutamate transport and excitotoxicity are involved in the pathogenesis of many MN diseases, as already well known for ALS (Rothstein et al., 1995). Such perturbations can also contribute to SMA disease, by enhancing MN death: thus, Riluzole and ceftriaxone, two different kind of drugs able to influence glutamatergic signaling and both FDA-approved for ALS (albeit with modest efficacy), have been proposed as repurposed compounds in clinical SMA trials. Riluzole exerts its anti-glutamatergic action, by enhancing the uptake of glutamate into astrocytes and by inhibiting its release blocking voltage-gated Na^+^ currents, thus preventing the neurotransmitter accumulation in the extracellular space and degeneration of MNs by excitotoxicity. When administered to *Smn*F7/F7; NSE-Cre mice SMA mice, Riluzole significantly attenuated disease progression (Haddad et al., 2003). Similarly, in a small phase I clinical study with enrolled SMA type I infants, Riluzole was proved to be safe and able to mitigate the natural course of the disease (Russman et al., 2003; Wadman et al., 2020). Although some aspects of Riluzole mechanism of action still need to be clarified in SMA, Dimitriadi and coll. proved that the drug acts on Ca^2+^-activated K channels, thus determining the improvement of MN functionality in several SMA models (Dimitriadi et al., 2013).

Ceftriaxone (a β-lactam antibiotic, also known as Rocephin) is used for the treatment of a number of bacterial infections (Koster, 1986; Schito and Keenan, 2005). Given its potential to reduce glutamate toxicity by modulating glial glutamate transporters (GLT1, EAAT2) (Calder et al., 2016; Ramalho et al., 2018), ceftriaxone efficacy has been tested in several MN disease models. Its administration in SMNΔ7 mice provided neuroprotective effects by modulating the glutamate transporter GLT1, the transcription factor Nrf2 and SMN protein levels, improving neuromuscular phenotype and increasing animal survival (Nizzardo et al., 2011). Therefore, also considering the safety and tolerability of ceftriaxone administration in ALS patients, the potential repositioning of β-lactam antibiotics as a treatment for SMA has been suggested (Nizzardo et al., 2011; Cudkowicz et al., 2014; Calder et al., 2016).

Another successful DR concerns the glutamate inhibitor Lamotrigine, commonly used for the treatment of various neuropsychiatric disorders and epilepsy. Its prolonged administration to adult type II and III SMA patients (Ng et al., 2008; Naguy and Al-Enezi, 2019) prevented the deterioration of motor functions for almost 5 years of treatment (Nascimento et al., 2010; Wadman et al., 2020).

Moreover, the imbalance in excitatory/inhibitory signaling have been also speculated in SMA. In particular, the enhancement of GABAergic neurotransmission in *C. elegans* SMA models was able to correct the locomotor dysfunctions (Wu et al., 2018). Moreover, in the work of Sleigh et al., a chemical library DS highlighted the rescuing of phenotypic dysfunction by Gaboxadol, a potent agonist of a specific extrasynaptic GABAA receptor subtype (Sleigh et al., 2011). Gaboxadol has been proposed for the treatment of insomnia (Mathias et al., 2005; Roth et al., 2010) and two neurological development disorders, Fragile X syndrome and Angelman syndrome (Identifier: NCT04106557), in which it improves behavioral and motor dysfunctions by enhancing GABAergic transmission (Cogram et al., 2019; Keary et al., 2020). Its repositioning for SMA treatment could be also suggested. Likewise, Gabapentin (Neurontin), a FDA-approved drug whit a molecular structure similar to GABA, acts by inhibiting calcium channels. Gabapentin is used for the treatment of different forms of neuropathic pain, for anxiety disorders and alcoholism (Field et al., 1997; Caraceni et al., 2004; Pfizer Inc., 2009; Moore et al., 2011; Levine et al., 2019). Considering the neuroprotective effect of gabapentin in nerve damage-induced chronic neuropathic pain (Wiffen et al., 2017), two different clinical trials were conducted enrolling SMA type II and III patients (Miller et al., 2001; Merlini et al., 2003). These trials confirmed a significant gabapentin-dependent improvements in the so-called “leg megascore” (calculated by summing knee flexion, knee extension and foot extension scores) and muscle strength, recommending further studies to evaluate prolonged administration of the drug in SMA patients (Merlini et al., 2003; Wadman et al., 2020).

### Neuromuscular Junction Stabilization

The NMJ represents the interaction core between the motor nerve terminal and the skeletal muscle fiber. SMN, neurotrophic factors (Stanga et al., 2016), “auxiliary proteins” (as neuregulins, dystrophin-glycoprotein complex, ErbB receptors, Wnts), miRNAs (as miRNA-9 and miRNA-206) and agrin (a heparan sulfate proteoglycan) seem to contribute to maturation and/or stabilization of NMJs (Valsecchi et al., 2015; Boido and Vercelli, 2016; Boido et al., 2018; Guarino et al., 2020). Given this evidence, DS and DR approaches have been proposed to improve NMJ development/stabilization in SMA patients.

To this purpose, Amifampridine, a FDA-approved drug used to treat Lambert-Eaton myasthenic syndrome, is a promising compound. Its efficacy has been also tested in Myasthenia Gravis (MG) patients: indeed, although Amifampridine acts on presynaptic terminal blocking K+ channels regulating ACh release, its positive effects were also observed in postsynaptic disorder such as MG (Bonanno et al., 2018). Since NMJs show both presynaptic and postsynaptic defects in SMA, Amifampridine can be considered a possible candidate to improve overall conditions of SMA patients (Hoolachan et al., 2019): to this aim, the recruiting-phase II clinical trial NCT03781479 is currently ongoing (Maddison et al., 1998; Hoolachan et al., 2019).

Similar effects are expected from Tideglusib, a glycogen synthase kinase 3 beta (GSK-3β) inhibitor, not yet FDA-approved, mainly investigated for AD and progressive supranuclear palsy treatments (Lovestone et al., 2015; Stamelou et al., 2016). However, Tideglusib was also proposed for a clinical trial on congenital myotonic dystrophy (Identifier NCT02858908, completed in January 2018) in whom excellent results were obtained with improvement in muscle growth (Horrigan et al., 2018); moreover, another trial is ongoing (Identifier NCT03692312). In addition, an image-based screening of chemical libraries showed that GSK-3β chemical inhibitors and short hairpin RNAs increase SMN protein levels and block cell death (Makhortova et al., 2011). Altogether, these data suggest that Tideglusib, likewise Amifampridine, could be a valuable drug candidate for SMA treatment (Hoolachan et al., 2019).

DS also allowed the development and identification of myostatin-follistatin modulators. Myostatin, a member of the transforming growth factor β (TGFβ) superfamily of growth factors, is primarily expressed by skeletal muscle cells, where acts as a negative regulator of muscle mass. Follistatin binds and inhibits myostatin and other members of the TGFβ family, contributing to the correct balance of muscle protein metabolism (Lee and McPherron, 2001). Dysregulation of myostatin-follistatin signaling pathway has been studied in several neuromuscular diseases, including SMA (Mariot et al., 2017; Shorrock et al., 2018). Myostatin expression is impaired in serum and muscle biopsies of SMA patients (Mariot et al., 2017), and its inhibition with intramuscular injection of AAV1-follistatin or muSRK-015P monoclonal antibody ameliorated muscle mass functions in different models of SMA mice (Feng et al., 2016; Long et al., 2019). Therefore, preventing myostatin activation has been widely suggested as therapeutic approach in SMA. To this aim, Scholar Rock developed several laboratory-made monoclonal antibodies against myostatin: by carrying out a phenotypic screening on a dexamethasone-induced muscle atrophy murine model, the SRK-015 antibody was proved as able to fully prevent muscle function loss (Pirruccello-Straub et al., 2018). Given this evidence, it has been tested in two groups of SMNΔ7 mice (which received different pharmacologic restoration of SMN, to reflect early or late therapeutic intervention) leading to positive effects by increasing muscle mass and function (Long et al., 2019). The efficacy of SRK-105 is currently evaluated in a phase 2 trial (TOPAZ, Identifier: NCT03921528) involving type II and III SMA patients (see Scholar Rock Reports First Quarter, 2020). Finally, another FDA-approved drug, successfully repositioned for SMA therapy, is Salbutamol (also known as albuterol; brand name Ventolin), widely used to reduce bronchospasm in some respiratory pathological conditions (i.e., asthma and chronic obstructive pulmonary disease). As a short-acting compound with selective agonist activity on β2-adrenergic receptors, Salbutamol has been evaluated for its possible beneficial effects on impaired SMA neuromuscular synaptic transmission and NMJ functions. It has been shown that Salbutamol determines a rapid and significant increase of FL-SMN mRNA and protein in SMA fibroblasts, predominantly by promoting exon seven inclusion in *SMN2* transcripts (Angelozzi et al., 2008): these results were further confirmed in peripheral blood leukocytes of SMA type II–III patients (Tiziano et al., 2010). Moreover, when administered to SMA type II and III patients, Salbutamol induced an overall improvement of motor performances and lung/inspiratory functions and a reduction of the perceived fatigue, suggesting further studies on the molecular mechanisms underlying these effects and its influence on muscles and NMJs (Pane et al., 2008; McCullagh et al., 2011; Giovannetti et al., 2016; Khirani et al., 2017; Frongia et al., 2019; Wadman et al., 2020).

## Conclusion

The traditional process for drug discovery (starting from preclinical testing to several clinical trial phases) is a time and money consuming procedure that, possibly, after 10–15 years could bring to a new potential molecule for the targeted disease (Kraljevic et al., 2004). In the last years, DS and DR became powerful strategies for finding new molecules and/or for giving a new birth to drugs already in use for other purposes. For sure, the development of potent computational approaches, the collection of omics data (genomics, proteomics or metabolomics) and the availability of databases, in combination with new biological experimental approaches, represent the key for DS and DR success and exploitation worldwide. Altogether, DS and DR represent a challenging field, which requires constant technological update but is extremely promising.

In this work, we reviewed both the models used for the screenings and the molecules identified by DS/DR in the context of SMA; exploring these approaches could represent a potential interesting market, especially for rare disorders such as SMA.

Moreover, interestingly, we highlighted that the molecules until now identified by DS/DR are involved and act on a limited number of molecular pathways (Figure 2). This indirectly contributes to shed light on the pathogenesis of SMA, clarifying which molecular cascades, organelles and cellular structures (including cell degradation, cytoskeletal dynamics, neurotransmitter and channel modulation) are particularly affected by SMN lack, and may represent therapeutic targets in combination or alternative to the *SMN*-dependent approaches.

Unexpectedly, pathways involving the transcription factor nuclear factor-κB (NF-κB), calpains and autophagy are not still be directly targeted by DR/DS studies, although they are known to be strongly related to SMA pathogenesis and their direct modulation already demonstrated to be effective. Indeed, NF-κB, it is known to play a fundamental role in the survival of neuronal cells (Bhakar et al., 2002) and is able to induce the release of different neurotrophic factors which are decisive for the survival of cultured MNs (Mincheva et al., 2011). In severe SMA mice, it has been observed that NF-κB is less expressed and its inhibition, by lentiviral delivery, causes the decrease of SMN protein levels (Arumugam et al., 2018). Calpains, a family of calcium-dependent proteases, are able to regulate the expression of SMN protein by direct cleavage. This has been observed both in muscle cells (Walker et al., 2008; Fuentes et al., 2010) and in MNs cells (de la Fuente et al., 2020): indeed, calpains’ activation induces SMN cleavage in MNs, while its knockdown or inhibition increases SMN level and prevents neurite degeneration. *In vivo*, the treatment with calpeptin, a calpain inhibitor, improves both lifespan and motor function of SMA mice (de la Fuente et al., 2019). Interestingly, calpain levels and activity are linked to autophagy, a finely tuned process which is fundamental for the maintenance of cellular homeostasis and which has already been observed as altered in SMA (Piras et al., 2017). Calpain reduction by lentiviruses in SMA cultured MNs can induce the expression of LC3-II, a well-known marker of autophagy (Periyakaruppiah et al., 2016). The activation of autophagy has also been observed after SMN reduction, underlining its important role in SMA pathophysiology (Garcera et al., 2013; Custer and Androphy, 2014). Although, current DS/DR studies did not yet identify molecules or drugs directly acting on NF-kB, calpain and autophagy.

Furthermore, the research field of biomarkers, besides supporting diagnosis it is important i) for evaluating the efficacy of new molecules, and ii) for revealing both specific and cross-disease pathological mechanisms: for this reason, it is largely exploited for neurodegenerative disorders such as SMA, ALS, and AD (Lanni et al., 2010; Stanga et al., 2012; Kariyawasam et al., 2018; Stanga et al., 2018a; Stanga et al., 2018b).

However, besides the positive aspects deepened in this review, it is evident that DS/DR methodologies still need to be further improved. Indeed, sometimes, the promising molecules identified in preclinical studies then fail to assure an equivalent efficacy in human patients or, if effective, can encounter difficulties in the patenting phase (Cagan, 2016; Mohs and Greig, 2017; Pushpakom et al., 2018): a higher methodological standardization together with more stringent parameters could further implement the validity and the success of these powerful screening approaches.

## Author Contributions

Conceptualization MB; writing original draft GM, DR, and SS; supervision SS and MB; review editing MB; funding acquisition MB. All authors have read and agreed to the published version of the manuscript.

## Funding

This work was supported by the AFM Telethon (project number 22346) to MB, and by Girotondo/ONLUS and SMArathon-ONLUS foundations.

## Conflict of Interest

The authors declare that the research was conducted in the absence of any commercial or financial relationships that could be construed as a potential conflict of interest.
